# The presence of benzene ring activating CoA ligases for aromatics degradation in the ANaerobic MEthanotrophic (ANME) archaea

**DOI:** 10.1128/spectrum.01766-23

**Published:** 2023-09-27

**Authors:** Wei-Wei Liu, Piaopiao Pan, Ning-Yi Zhou

**Affiliations:** 1 State Key Laboratory of Microbial Metabolism, Joint International Research Laboratory of Metabolic & Developmental Sciences, and School of Life Sciences & Biotechnology, Shanghai Jiao Tong University, Shanghai, China; Nanjing Institute of Geography and Limnology Chinese Academy of Sciences, Nanjing, Jiangsu, China

**Keywords:** ANaerobic MEthanotrophic archaea, aromatic degradation, phenylacetyl-CoA ligase, benzoyl-CoA ligase

## Abstract

Petroleum-source and black carbon-source aromatic compounds are present in the cold seep environments, where ANaerobic MEthanotrophic (ANME) archaea as the dominant microbial community mediates the anaerobic oxidation of methane to produce inorganic and organic carbon. Here, by predicting the aromatics catabolic pathways in ANME metagenome-assembled genomes, we provide genomic and biochemical evidences that ANME have the potential of metabolizing aromatics via the strategy of CoA activation of the benzene ring using phenylacetic acid and benzoate as the substrates. Two ring-activating enzymes phenylacetate-CoA ligase (PaaK_ANME_) and benzoate-CoA ligase (BadA_ANME_) are able to convert phenylacetate to phenylacetyl-CoA and benzoate to benzoyl-CoA *in vitro,* respectively. They are mesophilic, alkali resistance, and with broad substrate spectra showing different affinity with various substrates. An exploration of the relative gene abundance in ANME genomes and cold seep environments indicates that about 50% of ANME genomes contain PCL genes, and various bacteria and archaea contain PCL and BCL genes. The results provide evidences for the capability of heterotrophic metabolism of aromatic compounds by ANME. This has not only enhanced our understanding of the nutrient range of ANME but also helped to explore the additional ecological and biogeochemical significance of this ubiquitous sedimentary archaea in the carbon flow in the cold seep environments.

**IMPORTANCE:**

ANaerobic MEthanotrophic (ANME) archaea is the dominant microbial community mediating the anaerobic oxidation of methane in the cold seep environments, where aromatic compounds are present. Then it is hypothesized that ANME may be involved in the metabolism of aromatics. Here, we provide genomic and biochemical evidences for the heterotrophic metabolism of aromatic compounds by ANME, enhancing our understanding of their nutrient range and also shedding light on the ecological and biogeochemical significance of these ubiquitous sedimentary archaea in carbon flow within cold seep environments. Overall, this study offers valuable insights into the metabolic capabilities of ANME and their potential contributions to the global carbon cycle.

## INTRODUCTION

In the cold-seep ecosystems, the anaerobic oxidation of methane (AOM) coupled with sulfate reduction is the primary energetic and biogeochemical process, which is mediated by a consortium of ANaerobic MEthanotrophic (ANME) archaea and sulfate-reducing bacteria (1). During the AOM process in the cold seep environments, the ANME are the key players in the methane assimilation with sulfate as electron acceptors (1). ANMEs [ANME-1 (2) and ANME-2 (3)] oxidize methane using the reversed methanogenic pathway with different electron-transporting pathways. By the metabolism of methane assimilation, ANME possesses the potential ability to produce low-molecular organic acid acetate (4) and propanoate (1) as an alternative novel carbon flow supporting large autotrophic and heterotrophic bacterial populations existing in the cold-seep areas. It was also reported the presence of the petroleum-source polycyclic aromatic hydrocarbons through oil seeps (5), and black carbon-source aromatic compounds deposited from the surface, including aromatic acid, phenol, and naphthalene (6). These results in the accumulation of petroleum and sedimentary aromatic hydrocarbons in the deep-sea cold seep environments. Taken together, the presence of aromatics and the ANME as the key players in cold seep environments into consideration, we propose that ANME may be involved in the metabolism or utilization of aromatics, in addition to its chemoautotrophic lifestyle.

During the microbial metabolism of aromatics, different activation strategies are employed for aromatic hydrocarbons activation, such as phosphorylation, fumarate insertion, hydroxylation, carboxylation, and methylation (7). Indeed, CoA ligation involved in ring activation is one of the major approaches for aromatic degradation (8), even for the subsequent reactions after fumarate insertion, carboxylation, decarboxylation, and methylation strategies. For the CoA ligation approach, phenylacetic acid (PAA) (8) and benzoic acid (BA) (9) are two major substrates for the aromatic acid CoA ligases forming the central aromatic intermediates (10). The two aromatic acid CoA ligases, phenylacetate-CoA ligase (PCL) and benzoate-CoA ligase (BCL) catalyze the ligation of CoA to the aromatic acids producing phenylacetyl-CoA (PA-CoA) and benzoyl-CoA (BA-CoA). Then the two CoA thioesters are transformed into nonaromatic compounds by corresponding multicomponent epoxidases, resulting in the formation of acetyl-CoA and succinyl-CoA in the aerobic pathways (9, 11). For the anaerobic degradation pathway, PA-CoA is firstly oxidated forming BA-CoA (12) and the BA-CoA is then dearomatized by the multicomponent ATP-dependent (13) or ATP-independent (14) benzoyl-CoA reductase (BCR). Since the PCL and BCL are recruited in both aerobic (9, 11) and anaerobic (10, 11) aromatics degradation pathways (Fig. S1), the two aromatic acid CoA ligases activating the aromatic rings play a key role in the control of the potential precursors into the downstream degradation pathway.

In contrast to the extensive research on aromatic compounds degradation in bacteria, only few cases from archaea have been reported to degrade aromatic compounds, illustrated by halophilic archaea using dioxygenases (15) and hyperthermophilic archaea using BCR (16). A study on the deep-sea archaea to degrade the aromatic compounds showed that *Thermoprofundales* (Marine Benthic Group D archaea) was involved in the aromatics degradation through PAA via the CoA activation strategy in the deep-sea hydrothermal sediment (8). Nevertheless, no further studies have been reported on archaea from deep-sea cold seep, hampering us from understanding the role of archaea on aromatics degradation and acting as a possible microbial carbon pump in seep area.

To verify the hypothesis that archaea ANME in cold seep may be involved in the metabolism or utilization of aromatics, this study explored the presence of aromatic degradation genes in ANME genomes, the function and properties of PCL and BCL from ANME, and the relative abundance of the PCL and BCL genes in ANME genomes and cold seep environments. The potential of ANME to transform or utilize the aromatic hydrocarbons via the pathway involving PCL and BCL was explored by genomic analyses and enzymatic characterization. This study may provide evidences to elucidate that the dominant archaea ANME in cold seeps may be involved in the metabolism or utilization of aromatics apart from its chemoautotrophic lifestyle.

## RESULTS

### Putative PAA and BA pathway for aromatics degradation in ANME

**Fig 1:**
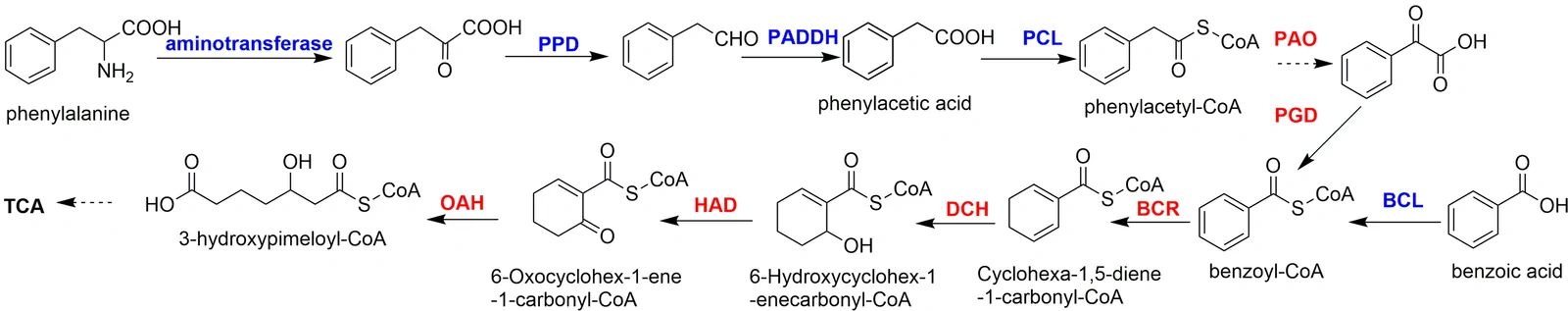
The potential aromatic compounds degradation pathway in ANME MAGs. Reconstructed aromatic compounds degrading pathway based on MAGs of ANME from the gulf of Cadiz. Enzymes identified in ANME MAGs are labeled in blue and those unidentified in any MAGs are labeled in red. Solid arrows indicate a single step was required to catalyze the reaction, while dotted arrows mean more than one step were required to catalyze the reaction. BCL, benzoate-CoA ligase; BCR, BCoA reductase; DCH, cyclohexa-1, 5-dienecarbonyl-CoA hydratase; HAD, 6-hydroxycyclohex-1-ene-1-carbonyl-CoA dehydrogenase; OAH, 6-oxocyclohex-1-ene-carbonyl-CoA hydrolase; PADDH, phenylacetaldehyde dehydrogenase; PAO, phenylacetyl-CoA:acceptor oxidoreductase; PCL, phenylacetate-CoA ligase; PGD, phenylglyoxylate dehydrogenase.

Based on the obtained five ANME MAGs from the Gulf of Cadiz (4), the genes involved in the aromatic degradation pathway were searched by BLASTp (the detailed information of the five ANME MAGs is in Table S1 in Supporting Information). As a result, the putative genes of PCL and BCL were identified in all the five genomes of ANME, but no single genes encoding monooxygenases and ring-cleavage dioxygenases were found. The major process and the involved enzymes accounting for the degradation of aromatic amino acid through the PAA and BA pathway were thus proposed as shown in Fig. 1 and each gene for the PAA and BA pathway distributed in the five MAGs is shown in Table S2 (in Supporting Information). Briefly, in this pathway, the aromatic amino acid phenylalanine is generally regarded to be firstly transformed to phenylpyruvate by aminotransferase. Then the carboxyl group of phenylpyruvate was hydrolyzed by the decarboxylase. Afterward, the formed phenylacetaldehyde was transformed to phenylacetate which is catalyzed by dehydrogenase (17). The phenylacetate was ligated to CoA to active the aromatic ring and the formed product phenylacetyl-CoA was proposed to be transformed to benzoyl CoA by a multicomponent oxidoreductase and a multicomponent dehydrogenase for the anaerobic living condition of ANME. Also, BCL found in ANME genomes can active aromatic ring with BA as substrate for being ligated to CoA. For the putative downstream pathway, the formed product benzoyl CoA can be dearomatized by multicomponent reductases (BCR), then the product was proposed to be hydrated, dehydrogenated, and hydrolyzed to open the ring (9).

### The aromatic acid activating enzymes PCL and BCL in ANME are functionally active

**Fig 2:**
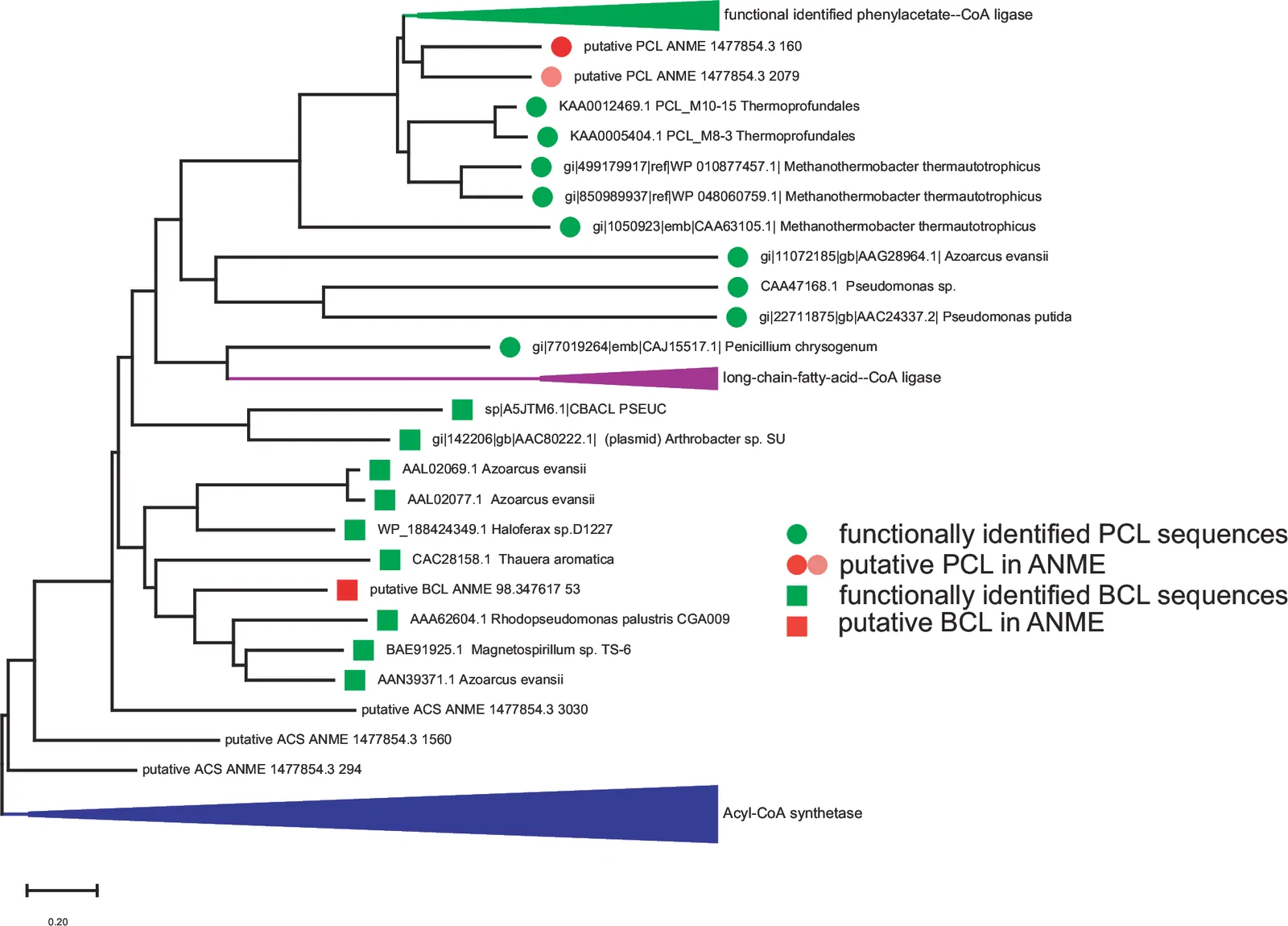
Phylogenetic analysis of candidate PCL and BCL encoding genes found in ANME MAGs. Phylogenetic trees showing the relationship between candidates PCLs and BCL from ANME and functionally defined PCLs and BCLs. Sequences of long-chain fatty acid CoA ligase and acetyl coenzyme A synthetase collapsed in the tree were obtained from the KEGG database.

Each ANME MAG from the Gulf of Cadiz contains at least one copy of PCL and BCL genes. Among these sequences, the BCLs from all five MAGs share the same sequence, but PCLs have two variants with 52% identity. The phylogenetic analysis using all the PCL and BCL sequences from the five ANME MAGs revealed that the putative PCLs from ANME were located in the branch of the functionally confirmed PCLs used in a previous study (8). These putative PCLs have approximately 45% identity with the PCLs of known function from terrestrial bacteria and hydrothermal sediment (Fig. 2). Also, the putative BCL from ANME was close to the functionally confirmed BCL or the CoA ligases for benzoic acid derivatives. The putative BCL shares approximately 31–42% identity with the functional BCLs from terrestrial bacteria.

**Fig 3:**
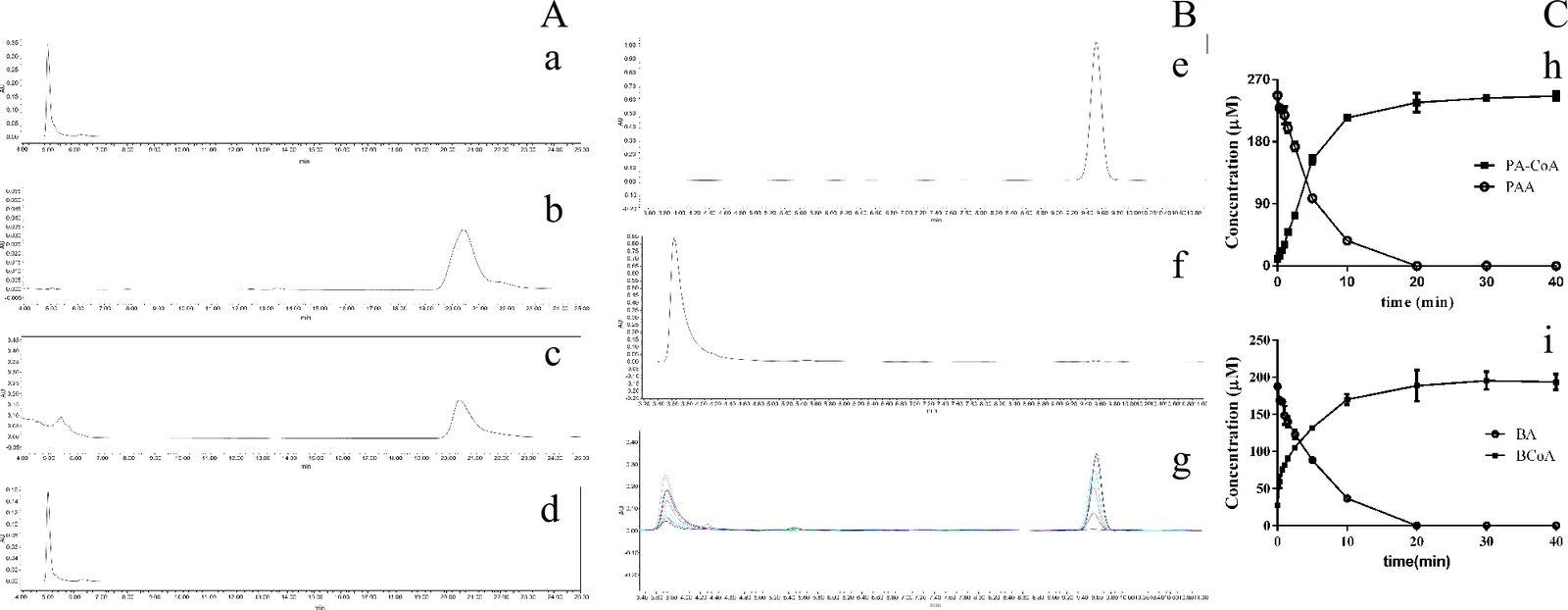
The functional identification of aromatic acid activating enzymes (PCL and BCL) in ANME. (**A**) The biochemical function confirmation of PaaK1_ANME_ and PaaK2_ANME_ (including a, **b, c, **d). (a) The peak of PAA; (b) the peak of PA-CoA; (c) the peaks of substrate and product in the reaction mixture catalyzed by PaaK1_ANME_; and (d) the peak of substrate and product in the reaction mixture catalyzed by PaaK2_ANME_. (**B**) The biochemical function confirmation of BadA_ANME_ by HPLC (including e, **f, **g): the peaks of BA (e), BA-CoA (f), and substrate and product during the time-course of the reaction mixture catalyzed by BadA_ANME_ (g) .(**C**) The time-course of reaction of transforming PAA to PA-CoA catalyzed by PaaK1_ANME_ (h) and transforming BA to BA-CoA catalyzed by BadA_ANME_ (i). PAA, PA-CoA, BA, and BA-CoA were quantified by HPLC.

To verify the biochemical functions of PCL and BCL from ANME, heterologous gene expression and activity assays for the two PCLs (PaaK1_ANME and_ PaaK2_ANME_) and one BCL (BadA_ANME_) was performed. The SDS-PAGE for the purified enzymes showed a band with an apparent molecular mass of 55 kDa for each enzyme (Fig. S2). For the enzymes assay, the products from the reaction catalyzed by PCL and BCL enzymes were detected at retention time of 20.5 min for PaaK1_ANME_ and 3.7 min for BCL by high-performance liquid chromatography (HPLC), corresponding to the retention time of PA-CoA (Fig. 3A) and BA-CoA, respectively (Fig. 3B). However, no activity was detected for the PaaK2_ANME_ (Fig. 3A through D, then the PaaK_ANME_ mentioned below refers to PaaK1_ANME_ (the accession number is MBC2699728.1). No product peak was detected in the reaction mixture without enzyme (Fig. S3). Time-course of the enzyme activity by HPLC analysis showed that PAA was stoichiometrically transformed to PA-CoA by PaaK_ANME_, and BA was stoichiometrically transformed to BA-CoA by BadA_ANME_ (the accession number is MBC2697931.1) (Fig. 3C). The specific activity of PaaK_ANME_ against PAA was 0.19 U/mg (Table 1), and that of BadA_ANME_ against BA was 9.49 × 10^−2^ U/mg (Table 2). These results revealed that both PCL and BCL from ANME were with enzymatic function in aromatic compound metabolism.

**TABLE 1:** The specific activities of PaaK_ANME_ to each substrate

Substrates	Specific activity (U/mg)
PAA	0.19 ± 0.01
2HPA	0.25 ± 0.02
3HPA	0.26 ± 0.02
4HPA	0.21 ± 0.01
3,4DHPA	0.15 ± 0.002

**TABLE 2:** The specific activity of BadA_ANME_ for each substrate

Substrates	Specific activity (10^−2^ U/mg)
BA	9.49 ± 0.23
2HBA	1.95 ± 0.03
3HBA	13.03 ± 2.01
4HBA	6.61 ± 0.12

### PCL and BCL in ANME are mesothermal and favor alkali condition with a broad substrate spectrum

**Fig 4:**
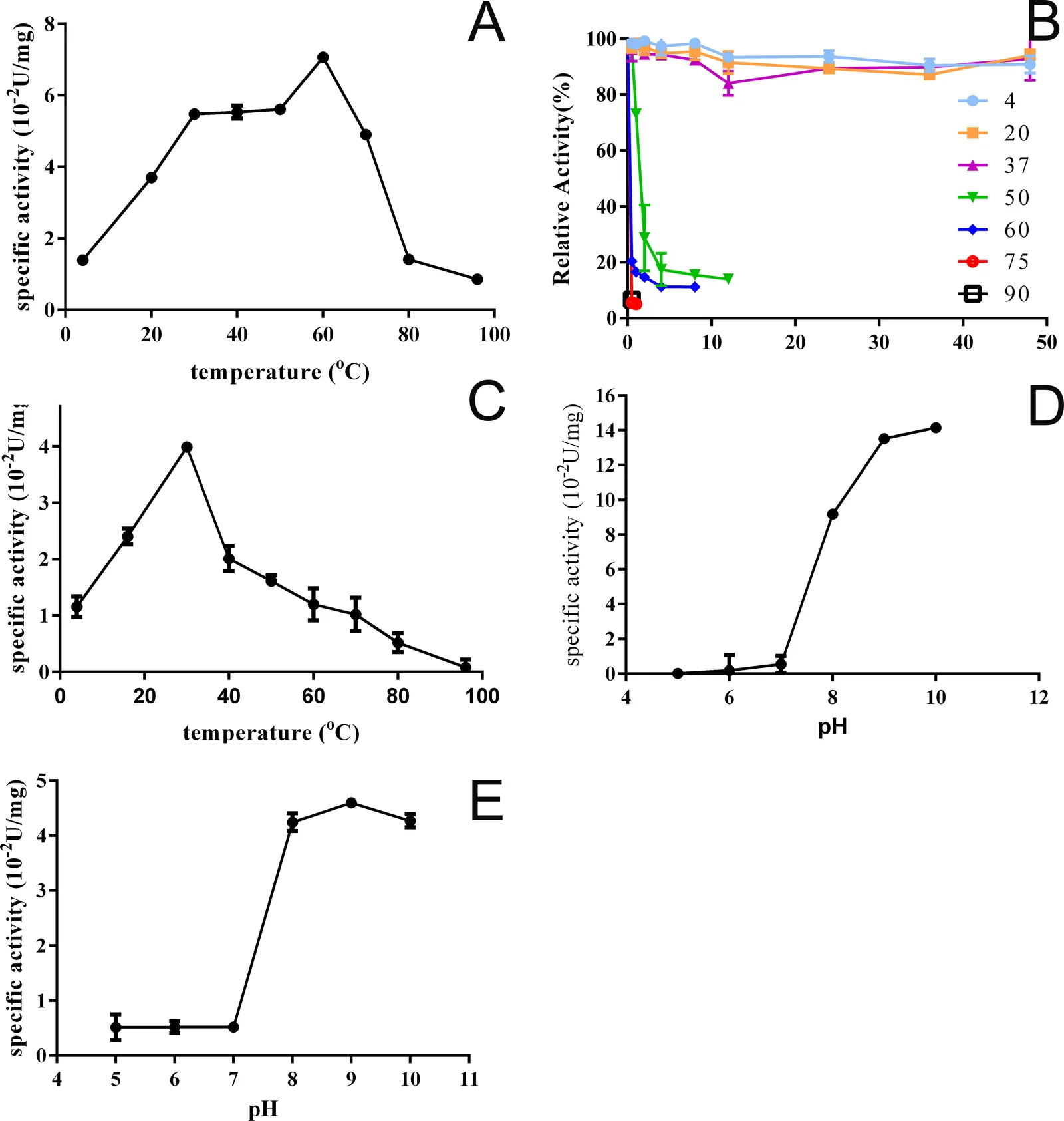
The PCL and BCL from ANME are mesothermal and alkali resistance. The optimum temperature of PaaK_ANME_ (**A**) and BadA_ANME_ (**C**). Enzyme activities of PaaK_ANME_ and BadA_ANME_ were assayed at different temperatures ranging from 4°C to 96°C. (**B**) The stability for temperature of PaaK_ANME_. Incubations were conducted in protein storage buffer (50 mM sodium-phosphate buffer, pH 7.6, 300 mM NaCl, and 10% glycerol) at the corresponding temperature. The enzyme activities of PaaK_ANME_ were assayed after 0.5 h, 1 h, 2 h, 4 h, 8 h, 12 h, 24 h, 36 h, and 48 h after the water bath. The activity of PaaK_ANME_ at the beginning without incubation was used as a control for 100% activity. The optimum pH of PaaK_ANME_ (**D**) and BadA_ANME_ (**E**). The activities of BadA_ANME_ and PaaK_ANME_ at the specific pH conditions were assayed. Tris-acetate (100 mM) was used as buffer covering pH 5–10. For the optimum temperature and optimum temperature assay, the concentration of substrate before and after enzyme reaction was measured using HPLC.

Although the aromatic acid activating enzymes (PCL and BCL) in ANME are proved to be functionally active, their adaption to the *in-situ* environments also required investigation. The PCL and BCL activities were determined at different temperatures ranging from 4°C to 96°C. As shown in Fig. 4A for the PaaK_ANME_, its highest activity exhibited at the temperature of 60°C but could achieve at least 50% of the highest activity in a broad temperature spectrum ranging from 20°C to 70°C. However, in the thermostability assay of the PaaK_ANME_, this enzyme was unstable when the temperature was higher than 37°C (Fig. 4B). The activities of the BadA_ANME_ were enhanced as the temperature increased up to 30°C, but decreased while the temperature higher than 30°C(Fig. 4C). It can be tentatively concluded that the PCL and BCL from ANME are mesothermal enzymes.

The optimal pH of PCL and BCL from ANME was also determined at different pH values ranging from 5 to 10 using 100 mM Tris-acetate buffer. Under these conditions, PCL and BCL from ANME achieved their highest activities at pH 10 and 9, respectively (Fig. 4D and E). PaaK_ANME_ and BadA_ANME_ from ANME retained less than half of and less than 10% of their highest activities at acid conditions (pH < 7), respectively. Overall, PaaK_ANME_ and BadA_ANME_ prefer alkali conditions for their activities and are very sensitive to acid conditions.

In addition to the environmental factors assay for their activity, the substrate spectra of PaaK_ANME_ and BadA_ANME_ from ANME were also explored. PAA and its derivatives, BA and its derivatives were used as the tested substrates for PaaK_ANME_, and BadA_ANME_, respectively. As shown in Table 1, the specific activities of the PaaK_ANME_ to PAA and different hydroxyl substituted derivatives were very similar, ranging from 0.15 to 0.26 U·mg^−1^. PaaK_ANME_ exhibited a similar kinetic parameter with *K*_*m*_ values ranging from 40.1 to 180.6 µM, and *k*_cat_ values ranging from 5.9 to 15.2 min^−1^ against PAA, 2-hydroxyphenylacetic acid, 3-hydroxyphenylacetic acid, 4-hydroxyphenylacetic acid, and 3,4-dihydroxyphenylacetic acid (Table 3). As for the BadA_ANME_, it exhibited relatively higher activities against BA, 3-hydroxybenzoate and 4-hydroxybenzoate ranging from 6.6 to 13 × 10^−2^ U·mg^−1^ but a lower activity (2 × 10^−2^ U·mg^−1^) for 2-hydroxybenzoate and no activity for 2-aminobenzoate (data not shown). The kinetic measurement showed that BadA_ANME_ possessed similar affinities and activities to BA, 3-hydroxybenzoate and 4-hydroxybenzoate with the *K*_*m*_ values ranging from 13 to 77 µM and *k*_cat_ ranging from 6.5 to 13.2 min^−1^ in the same order of magnitude (Table 4).

**TABLE 3:** Summary of *K_m_* and *k*_cat_ of PaaK_ANME_ for different substrates

Substrates	*K_m_* (μM)	*k*_cat_ (min^−1^)
PAA	180.6 ± 32.18	9.198 ± 0.4532
2HPA	40.06 ± 3.882	13.22 ± 0.2813
3HPA	68.87 ± 9.425	15.21 ± 0.5372
4HPA	49.15 ± 12.54	8.881 ± 0.5286
3,4DHPA	42.80 ± 15.03	5.857 ± 0.4330

**TABLE 4:** Summary of *K_m_* and *k*_cat_ of BadA_ANME_ for different substrates

Substrates	K_m_ (μM)	k_cat_ (min^−1^ RT)
BA	12.98 ± 1.563	6.762 ± 0.1513
3HBA	76.97 ± 14.69	13.18 ± 0.6763
4HBA	55.08 ± 6.910	6.517 ± 1.778
2HBA	–^*a*^	–^*a*^

^*a*^ –, not detected.

Based on these characteristics of PaaK_ANME_ and BadA_ANME_, it can be inferred that ANME may have capabilities of transforming or utilizing various aromatics in extremely energy-limited conditions in deep-sea cold seep environments.

### The wide distribution of PCL and BCL genes in ANME and cold seep environments

As enzyme identification above has suggested that the PCL and BCL played a key role in the control of potential precursors of PAA and BA into their downstream degradation pathway in five ANME MAGs from the Gulf of Cadiz, the universal presence of PCL and BCL genes in available ANME genomes is worth investigation. As many as 62 MAGs of ANME found in NCBI database were used to analyze the PCL and BCL gene distribution (the details for the 62 ANME MAGs are shown in Supplementary Tables). The PCL and BCL sequences were searched in ANME MAGs using the BLASTp, revealing that 29 MAGs harbor 47 copies PCL genes, but as few as 6 copies BCL genes were retrieved which are all from the 5 MAGs in this study.

To broadly explore the distribution of genes encoding aromatic acid activation in cold seep environments, the PCL and BCL genes were searched in a total of 77 cold seep environments (the details of the 77 cold seep environments are in Table S3 in Supporting Information). As many as 6,527 PCL sequences were found in metagenomic data sets from 77 cold seep environments (the detailed information for the 6,527 PCLs is in Supplementary Tables). On the other hand, 316 BCL sequences in total were found in a variety of bacteria and some archaea, indicating a wide distribution of aromatics metabolic potential among cold seep microorganisms (and the detailed information for the 316 BCLs is in Supplementary Tables).

**Fig 5:**
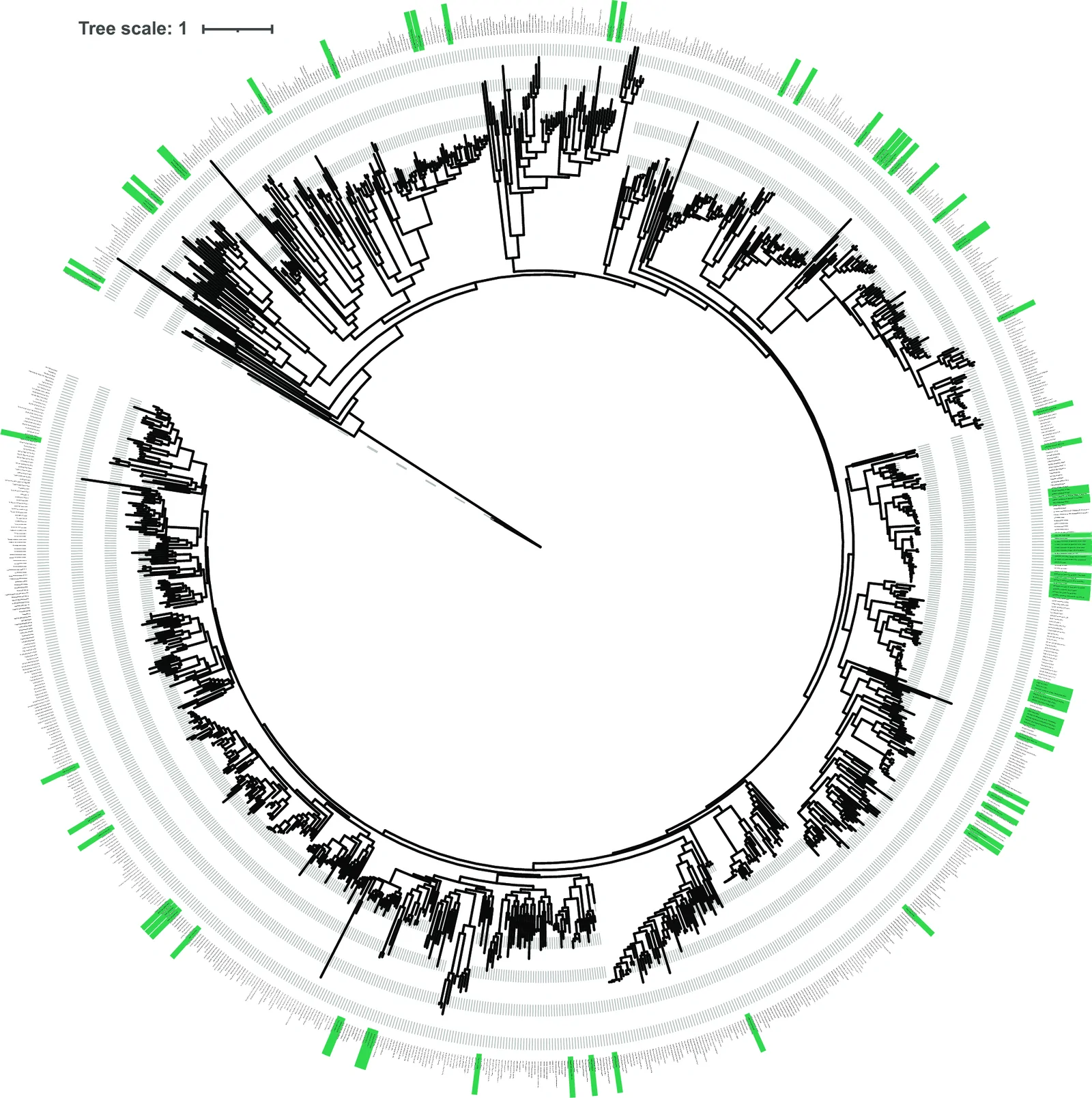
Distribution of PCL among archaea from cold seeps. Phylogenetic analysis of a total of 1,027 archaeal PCL sequences identified from 77 metagenomic data sets of cold seep samples. PCLs from ANME in the phylogenetic tree were highlighted in green.

**Fig 6:**
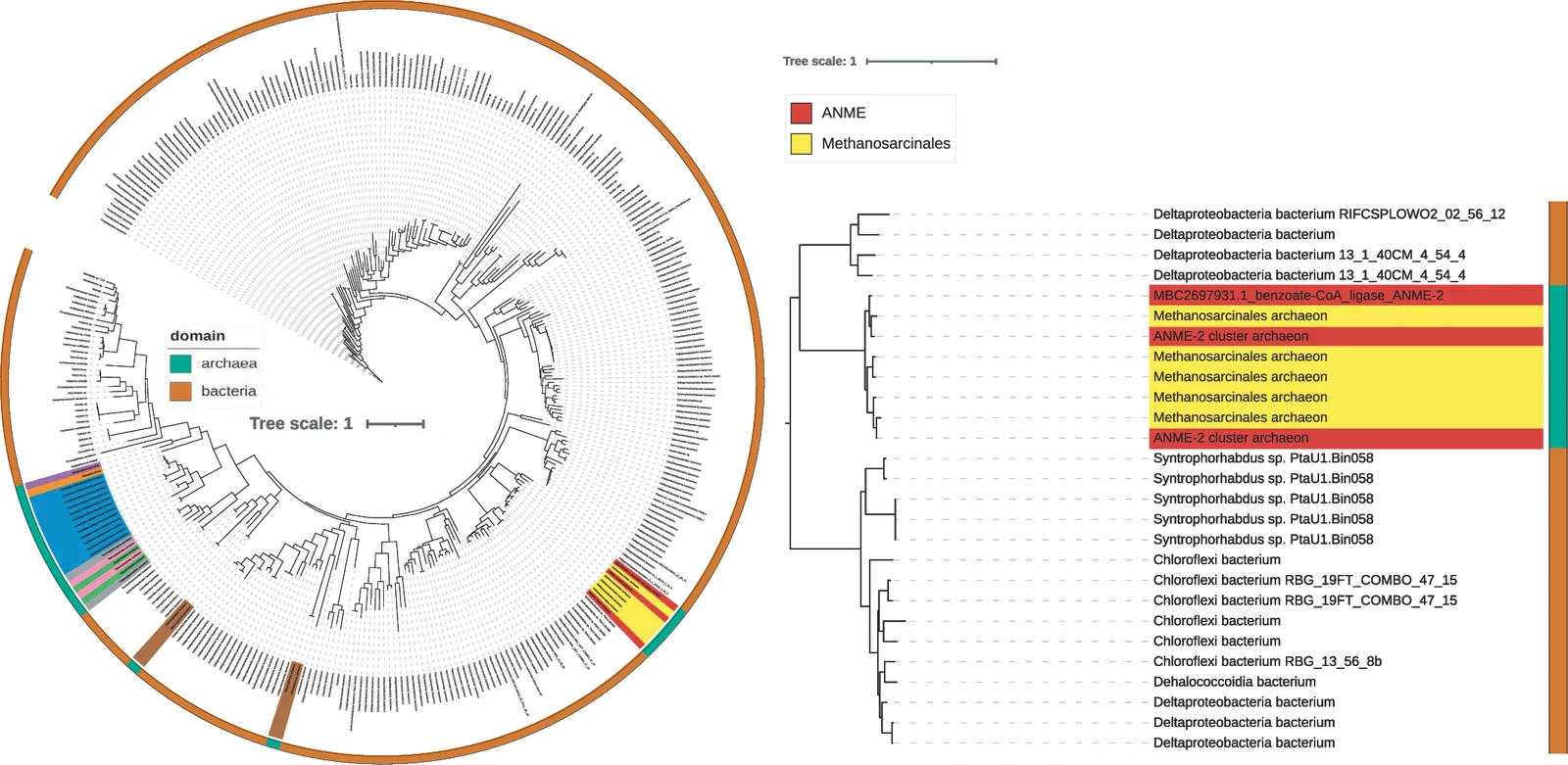
Distribution of BCLs from cold seeps. Phylogenetic analysis of a total of 316 BCL sequences from archaea and bacteria identified from 77 metagenomic data sets of cold seep samples. BCLs from ANME were highlighted in red.

Among the 6,527 PCL sequences, 1,027 sequences were from archaea and 59 sequences were from ANME. The archaea containing PCL genes mainly include ANME, *Archaeoglobales*, *Bathyarchaeota*, *Thermoplasmatales*, *Methanosarcinaceae*, *Methanophagales*, *Methanoregulaceae*, *Methanomicrobiales*, and *Lokiarchaeota* archaea. In particular, PCLs from archaea are not located in respective clusters according to the archaeal taxonomy but dispersive on phylogenetic tree, suggesting that PCL from each archaeon was not co-evolved with the archaeal taxonomy. This dispersiveness may be caused by the two copies of PCL sharing relatively moderate identity of 50% in ANME. PCLs from ANME clustered with those from *Thermoplasmata* (including *Thermoplasmatales*), *Methanophagales*, *Methanosarcinales* (including *Methanosarcinaceae*), and *Archaeoglobi* (including *Archaeoglobus fulgidus*, a sulfate reducing archaea) (Fig. 5). Among the 316 BCLs, 30 were from archaea including haloarchaeon, *Bathyarchaeota*, *Nitrososphaeria*, *Thermoplasmata*, *Methanosarcinales*, and ANME. The 30 BCL sequences from archaea are located in respective clusters according to the archaeal taxonomy on phylogenetic tree (Fig. 5). Two BCL sequences from ANME along with the function-identified BadA_ANME_ in this study were clustered with those from *Methanosarcinales* and adjacent to a clade of BCLs from bacteria Deltaproteobacteria and those from *Syntrophorhabdus* and *Chloroflexi* (Fig. 6). The different occurrence frequency of these two CoA ligases suggested a more important role of archaea in activating aromatic acids using PAA and its derivatives as substrates compared to using BA as substrate.

## DISCUSSION

ANME was usually considered to act as chemoautotrophs to oxidize methane (3) or produce acetate (4) or propanoate (1) supporting heterotrophic bacterial populations in the cold seeps in the previous reports. In this study, the presence of enzymatically active PCL and BCL proteins in the MAGs of ANME archaea tentatively indicated that ANME may utilize the strategy of the CoA ligation to active the benzene ring and probably then transform or degrade the aromatic compounds ultimately. The results in this study suggest that, apart from the methane oxidation and production of low-molecular organic acid as an autotrophic microorganism, ANME may be involved in the heterotrophic aromatic compounds metabolism or help some heterotrophic microorganisms to transform aromatics in the cold spring environment. The functional genes are actually PCL gene. Nevertheless, expression pattern condition in ANME *in vivo* was to be studied further. On the other hand, because the ANME population are commonly regarded as uncultivated and to form syntrophic aggregates with sulfate-reducing bacteria (3), it is generally accepted that it is not practical to enrich or isolate the ANME population for performing *in vivo* assays. Thus, the enzymatic assay is virtually the best evidence to support the metabolic potential of ANME. The presence of genes involved in the PAA and BA pathway for the aromatic degradation, and the absence of monooxygenase and ring-cleavage dioxygenase genes in ANME genomes, along with the anaerobic methanotrophic character of ANME implied that several aromatic compounds may be transformed or utilized by ANME without the participation of oxygen. To take it a step further, instead of activating the benzene ring using the introduction of hydroxyl groups by oxygenation and the substrate ring-cleavage by dioxygenation reaction, the aromatics transformation by ANME may be through the route of aromatic CoA thioesters. For the strategy of the ring activation by CoA ligation, the formed PA-CoA or BA-CoA could be degraded through epoxidation pathway (9, 11) or reductive pathway (9, 12). In a study regarding the uncultured *Thermoprofundales* archaea from hydrothermal sediment, genomic and transcriptomic evidences established that aromatic degradation in *Thermoprofundales* was using the PCL and BCR enzymes through the reductive pathway (8). But in ANME, no BCR or epoxidases encoding genes were found in the aromatic degradation pathway in this study.

The broad substrate spectra of Paak_ANME_ and BadA_ANME_ observed in this study could enable ANME to effectively adapt to benthic sediment environments under various carbon substrate conditions, especially in the oligotrophic pelagic ecosystem in the Gulf of Cádiz (18). Similarly, the PCLs from the uncultured archaea *Thermoprofundales* were also with broad substrate spectrum (8). But this was different for the PCLs from the terrestrial bacteria strain *B. cenocepacia* J2315 (8) and the thermophilic bacterium *Thermus thermophilus* strain HB27 (19), both with a much narrower substrate spectra. On the other hand, many BCLs reported did not accept monohydroxybenzoate isomers as substrates but usually use 2-aminobenzoate. And the reported *K*_*m*_ values of the BCLs were less than 50 µM ranging from 0.6 µM to 45 µM (20). In contrast, the BadA_ANME_ in this study has a similar affinity but uses monohydroxybenzoate isomers instead of 2-aminobenzoate.

The PCL gene was widely distributed in ANME genomes for about half of ANME MAGs containing the PCL gene. It might be more than 50% presence in ANME MGAs of PCL gene if considering the incompleteness of many metagenome-assembled genomes. And it could get different results if we have more ANME MAGs. The PCL and BCL genes were identified in many bacteria and archaea in 77 cold seep metadates, indicating a wide distribution of aromatic metabolism in cold seep microbes. The ubiquitous distribution of PCL gene was assayed in the previous research (8). The more frequent distribution of the PCL gene than BCL in ANME genomes and metadata may indicate that the precursor aromatic compounds in cold seep environments may be more likely ring-activated via phenylacetate. The close relationship between BCLs from ANME and *Methanosarcinales* archaea suggested their common evolutionary origin in aromatics metabolism, further indicating the close phylogenetic relation between *Methanosarcinales* and ANME-2 described previously (3).

It was reported that *Chloroflexi* (*Dehalococcoidia*) and *Deltaproteobacteria* in the cold seep area potentially degrade aromatic hydrocarbons using benzoyl-CoA as central metabolite but without BCL in some MAGs (21), the BCL genes in ANME may well help these bacteria to process the transformation of benzoate. On another hand, all the five ANME MAGs contain the putative MFS-type benzoate permease (with the identity of about 30% with the functionally identified benzoate transporter BenK), further enhancing the possibility of the transformation of BA by ANME in the cold seep environments.

In conclusion, this study has not only enhanced our understanding of the nutrient range and metabolic repertoire of ANME but also helped to explore the additional ecological and biogeochemical significance of this ubiquitous sedimentary archaea ANME in the carbon flow in the cold seep environments.

## MATERIALS AND METHODS

### Sample description and metagenomic information

The sampling description, sample incubation procedure, metagenomic performance, and MAGs information in this study were described previously (4). The constructed five MAGs for ANME were used in this study (4).

### Gene identification

Based on the obtained five ANME MAGs from the Gulf of Cadiz (4), the genes involved in the aromatic degradation pathway were searched by BLASTp (see Table S1 in Supporting Information for the detailed information of the five ANME MAGs), particularly the genes encoding enzymes for aromatic amino acids degradation. As mentioned in the Introduction section, the strategy of hydroxyl activation uses oxygenases, whereas the strategy of CoA ligation for the ring activation uses CoA ligases. For the latter strategy, PCL and BCL are the key enzymes controlling the precursor substrates into the downstream degradation pathway. Therefore, monooxygenases, dioxygenases, and ligases were used as reference sequences to perform BlastP. The amino acid sequences of genes encoding ring-cleavage dioxygenases (including catechol 1, 2-dioxygenase, catechol 2, 3-dioxygenase, gentisate 1, 2-dioxygenase, protocatechuate 3, 4-dioxygenase, and protocatechuate 4, 5-dioxygenase) and genes involved in the PAA and BA pathway (including amino transferase, phenylpyruvate decarboxylase, phenylacetaldehyde dehydrogenase, PCL, phenylacetyl-CoA oxidoreductase, phenylglyoxylate dehydrogenase, BCL, benzoyl CoA reductase, cyclohexa-1,5-dienecarbonyl-CoA hydratase, 6-hydroxycyclohex-1-ene-1-carbonyl-CoA dehydrogenase, and 6-oxocyclohex-1-ene-carbonyl-CoA hydrolase) were obtained from the KEGG GENES Database. Amino acid sequences were consequently BLASTp against the local database (*e* value < 1e−10, identity > 40%, and aligned length > half of the hit sequences) to search for candidate genes. All of the blast hits were then double-checked by BLASTp searching against the NCBI NR database. PCL and BCL in 62 ANME MAGs were also identified using the BLASTp method mentioned above.

### Bacterial strains, plasmids, growth conditions, and chemicals

The bacteria strains and plasmids used in this study are listed in Table S4. *Escherichia coli* strains carrying and expressing the cloned genes were cultivated at 37°C in lysogeny broth (LB) medium supplemented with 50 µg/mL kanamycin. Analytically pure chemical PAA was purchased from Sinopharm Chemical Reagent Co., Ltd. (Shanghai, China), the PAA derivatives from Aladdin Biochemical Technology Co., Ltd. (Shanghai, China) including 2-hydroxyphenylacetate (2HPA), 3-hydroxyphenylacetate (3HPA), 4-hydroxyphenylacetate (4HPA), 3,4-dihydroxyphenylacetate (3,4DHPA), as well as BA and its derivatives 2-hydroxybenzoate (2HBA), 3-hydroxybenzoate (3HBA), 4-hydroxybenzoate (4HBA), 3,4-dihydroxybenzoate (3,4DHBA), and phosphoenol pyruvate. The CoASH and ATP were obtained from Sangon Biotech Co., Ltd. (Shanghai, China), and the PA-CoA from Sigma Chemical (St. Louis, MO, USA). The biochemical reagents myokinase, pyruvate kinase, and lactate dehydrogenase were all purchased from Sigma Chemical.

### Expression and purification of proteins

PCL-encoding genes were synthesized in *E. coli* codon usage by Yidao Biotechnology Co., Ltd. (Nanjing, China). The gene encoding the BCL using the *E. coli* codon usage was synthesized by Shanghai RealGene Biotech. The PCL and BCL genes were respectively cloned into the expression vector pET-28a between the sites *Nde*I/*Bam*HI and *Bam*HI/*Xho*I. The PCL and BCL proteins were heterologously overexpressed in *E. coli* BL21 (DE3) strain by the induction of 0.2 mM IPTG at 16°C for 16 h in a total volume of 1.0 L when the OD_600nm_ reached 0.5. The harvested cells were washed two times with washing buffer (50 mM phosphate buffer pH 7.6), and then lysed by sonication in lysed buffer (50 mM phosphate buffer pH 7.6, 200 mM NaCl, and10% glycerol). The cell lysate was centrifuged at 19,000 × *g* for 45 min to remove the debris, after which the supernatant was collected and applied to a Ni-NTA column using the AKTA system. After washing with the buffer (50 mM phosphate buffer pH 7.6, 200 mM NaCl, 10% glycerol, and 20 mM imidazole), the his-tagged protein was eluted with the elution buffer (50 mM phosphate buffer pH 7.6, 200 mM NaCl, 10% glycerol, and 250 mM imidazole), and then dialyzed in the storage buffer (50 mM phosphate buffer pH 7.6, 200 mM NaCl, and 10% glycerol). An empty pET-28a vector was used as a control. All chemicals used here were reagent grade and were purchased from Sinopharm Chemical Reagent Co., Ltd.

### Standard enzyme assay

The standard enzyme reaction mixture contained 50 mM Tris/HCl (pH 8.0), 10 mM MgCl_2_, 5 mM CoA, 5 mM ATP, 5 mM PAA or BA as substrate, and a suitable amount of purified enzyme. The mixture without PCL/BCL enzyme or without PAA/BA substrate as the control assay. Neither of the two control mixtures showed enzymatic activity. The enzymatic reaction was carried out at room temperature. The substrates and products in the assay were determined using HPLC. A time-course assay was processed at 5 mM MgCl_2_, 0.2 mM PAA, 2 mM CoA, and 1 mM ATP at 50℃. The reactions were started by the addition of enzyme and stopped by adding one volume of methanol or 1/20 volume of formic acid. Finally, the substrate and product were quantified with standard curves using HPLC. One unit of enzyme activity is expressed as the consumption of 1 µmol of substrate in 1 min in this study. Specific activities are expressed as units per mg of protein.

### The optimum temperature and the thermostability assays of enzymes

For the temperature assay of BCL, the standard enzyme assay mixture was used, where the assay mixtures were kept in a water bath at the corresponding temperatures of 4°C, 20°C, 30°C, 40°C, 50°C, 60°C, 70°C, 80°C, and 96°C for 30 min. The substrate concentration was quantified with standard curves using HPLC. Then the enzyme activities in different temperatures were calculated according to the decrease of the substrate amount. The optimum temperature for PCL activity and its thermostability were determined using the hydroxamic acid assay. The assay mixture contained 50 mM Tris–HCl (pH 8.0), 10 mM MgCl_2_, 10 mM PAA, 5 mM ATP, 2.5 mM CoA, and 50 µL hydroxylamine in a reaction mixture with a total volume of 250 µL. After 30 min in a water bath at the corresponding temperature, 450 µL ferric chloride was added to stop the reaction. The assay mixture was then kept on ice for 30 min and centrifuged in an Eppendorf 5810R microcentrifuge for 2 min, after which the absorbance at 540 nm was measured at room temperature using a PerkinElmer Lambda 25 UV–VIS spectrometer in a 1.0 × 1.0 cm^2^ quartz cuvette. The extinction coefficient of phenylacetyl hydroxamate under these conditions was 900 /M·cm (22). For the thermostability assay of PCL, the purified enzyme was in water bath at different temperatures for hours to tens of hours. Then the enzyme activities in different temperatures were determined using the hydroxamic acid assay, and the activities relative to the initial activity without water bath were calculated.

### Characterization of optimum pH for PCL and BCL enzymes

For the optimum pH assay of BCL and PCL, the standard enzyme assay mixture in the 100 mM Tris/acetic acid buffer with pH ranging from 5 to 10 was used. The substrate concentration was quantified with standard curves using HPLC. Then the enzyme activities in different pH were calculated according to the decrease of the substrate amount.

### Kinetic measurement of enzymes

The apparent *K*_m_ and *k*_cat_ values of PCL for PAA along with its derivatives and BCL for BA along with its derivatives were determined using a coupled enzyme assay in which myokinase, pyruvate kinase, and lactate dehydrogenase were used to measure the AMP or ADP formation at room temperature (23). The reaction mixture (500 µL) contained 50 mM Tris–HCl (pH 8.0), 10 mM MgC1_2_, 1 mM ATP, 0.5 mM CoA, 0.4 mM NADH, 1 mM phosphoenol pyruvate, myokinase (17 nkat), pyruvate kinase (17 nkat), and lactate dehydrogenase (25 nkat) with increasing concentrations of each substrate (for BA and its derivatives from 2.5 to 1 mM, for PAA and its derivatives from 5 to 1.5 mM) and purified enzyme (102.8 µg protein for BCL and 52 µg protein for PCL). The decrease in absorbance at 340 nm was measured at room temperature with a PerkinElmer Lambda 25 UV–VIS spectrometer in a 1.0 × 1.0 cm^2^ quartz cuvette. The stoichiometry of 1 and 2 mol NADH oxidized per mole of aromatic acid was taken as evidence of ADP and AMP formation, respectively. The molar extinction coefficient of NADH was 6200 /M·cm at 340 nm (24).

### Analytical technique

PAA and BA were determined using an Agilent ZORBAX SB-C18 column (80 Å, 5 μm, 4.6 × 250 mm^2^) connected to Waters e2695 pumps and a Waters 2998 PDA detector set at 210 and 231 nm, respectively. The PAA substrate was eluted isocratically using 87% 50 mM phosphate buffer pH 6.4 (solution A), 9% methanol (solution B), and 7% acetonitrile (solution C) at a flow rate of 1 mL/min. The BA substrate was eluted isocratically using 80% 50 mM NH_4_Ac pH 4.2 (solution A), and 20% acetonitrile (solution B) at a flow rate of 1 mL/min. Typical retention times were 7.0 min for PAA and 8.6 min for BA under above conditions.

### Taxonomic identification of genes

To examine the distribution of BCL and PCL genes among archaea from cold seeps, 77 cold seep metagenomic data sets were collected. If the data set did not have available annotations in the IMG database, raw reads were firstly quality filtered using fastp (v0.23.2) (25), and filtered reads were individually assembled using MEGAHIT (v1.2.9) (26). For each assembly, protein-coding gene sequences and corresponding amino acid sequences were predicted using Prodigal (v2.6.3) (27) with the “-meta” setting. A total of 316 putative BCL sequences and 6,527 PCL sequences were identified using Hidden Markov Model profiles retrieved from KOfam(28) (accession numbers were shown in Supplementary Tables). The taxonomy of each protein sequence was determined by NR database with DIAMOND (v2.0.15.153) (29).

### Phylogenetic tree construction

PCL and BCL sequences from 77 cold seep environments metagenomic data sets were collected. The BCL sequences from cold seep environments metagenomic data sets along with those in ANME-2 from the Gulf of Cadiz in this study were aligned using Muscle v5.1 (30), after which columns were trimmed with trimAL v1.4.1 (31) using gap threshold 0.9 and cons 60. A phylogenetic tree was constructed by IQ-TREE (v2.2.0.3) (32) with LG + I + G4 model searched by ModelFinder (33) with 1,000 bootstraps. The alignment and the phylogenetic tree construction for archaeal PCL sequences from 77 meta data sets use the same method as BCL sequences as above except with LG + R10 models and 1,000 ultrafast bootstraps (34).

## SUPPLEMENTAL MATERIAL

Figures S1-S3，Tables S1-S4 spectrum.01766-23-s0001.docx Please see the file for details.

Supplementary Tables spectrum.01766-23-s0002.xlsx Sheets 1, 2, and 3.

ASM does not own the copyrights to Supplemental Material that may be linked to, or accessed through, an article. The authors have granted ASM a non-exclusive, world-wide license to publish the Supplemental Material files. Please contact the corresponding author directly for reuse.
