# Supplementary material for: The presence of benzene ring activating CoA ligases for aromatics degradation in the ANaerobic MEthanotrophic (ANME) archaea
Source: Microbiol Spectr. 2023 Sep 27;11(5):e01766-23. doi: 10.1128/spectrum.01766-23 (PMC10581246; doi:10.1128/spectrum.01766-23)
Supplement: Figures S1-S3，Tables S1-S4 — Please see the file for details. [file spectrum.01766-23-s0001.docx]

**Supplemental Material**

**The presence of** **benzene ring** **activating CoA ligases for aromatics degradation in the** **ANaerobic MEthanotrophic archaea (ANME)**

**Running title:** Benzene ring activating CoA ligases in ANME

Wei-Wei Liu, Piaopiao Pan, Ning-Yi Zhou*

State Key Laboratory of Microbial Metabolism, Joint International Research Laboratory of Metabolic & Developmental Sciences, and School of Life Sciences &

Biotechnology, Shanghai Jiao Tong University, Shanghai, 200240, China.

^*^Corresponding author: Ning-Yi Zhou

Email: ningyi.zhou@sjtu.edu.cn

Mailing address: Life Sciences Building, 800 Dongchuan Road, Shanghai Jiao Tong University, Shanghai 200240, China Tel.: 021-34204135. Fax: 021-34208261.



Figure S1. Many aromatic compounds can be degraded through BA (A) and PAA (B). (A) The BA precursory substrates include benzene, phenol, toluene, cresols, ethylbenzene, *p*-phthalic acid and biphenyl in the left of the figure. Benzoate-CoA ligase (BCL, in red and bold) catalyzes the reaction transforming benzoate to benzoyl-CoA (in black and bold). Some substrates can be precursor substance of benzoyl-CoA including 4-hydroxybenzoic acid, 4-aminobenzoic acid, phenylacetate and phenylpropionic acid using their own ligases. Then the formed benzoyl-CoA was catabolized through aerobic (the related genes are in red) and ATP-dependent (the related genes are in blue) or ATP-independent (the related genes of are in green) anaerobic pathway. (B) Many aromatic compounds in the left of the figure including aromatics with an even-number side chain (in purple), aromatic amino acids (in black), phenylpropane compounds (in blue) and contaminants phenylethanol and styrene (in red) were reported to be transformed to phenylacetate. Phenylacetate-CoA ligase (PCL, in red and bold) catalyzes the reaction transforming phenylacetate to phenylacetyl-CoA (in black and bold). Then phenylacetyl-CoA was catabolized through aerobic (the related genes are in red) and anaerobic (the related genes are in blue) pathway.


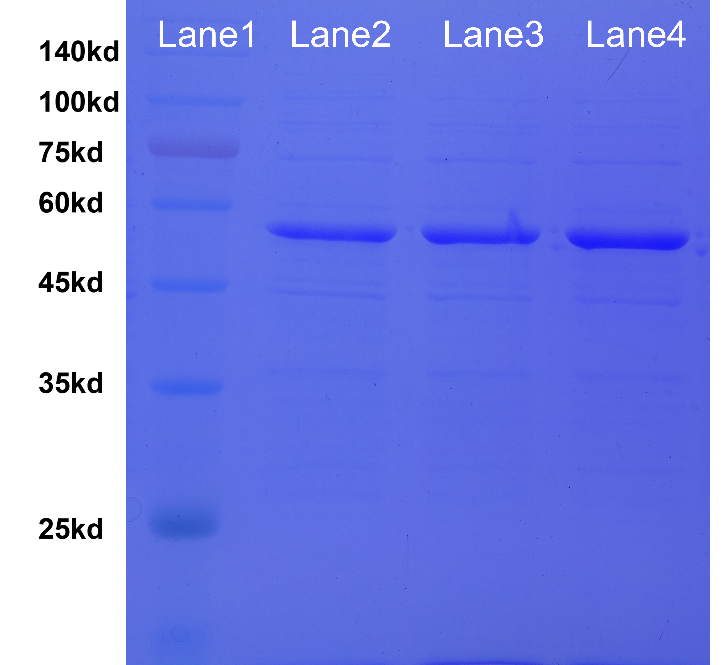


Figure S2. SDS-PAGE of the two purified overexpressed PCL and BCL proteins from ANME in *E*. *coli*. Lane1, the marker; Lane2, PCL1 from ANME; Lane3, PCL2 from ANME; Lane4, BCL from ANME.

Figure S3. HPLC traces for the reaction mixture without enzyme as negative control. The HPLC for the reaction mixture without Paak_ANME_ (A) or BadA_ANME_(B) as the negative control.

A

B

Table S1 Overview of ANME-2a metagenome assembled genomes used in this study^*^.

| **Bin_ID** | **Completeness (%)** | **Contamination (%)** | **G+C (%)** | **Contigs** | **N50** | **Size (M)** |
| --- | --- | --- | --- | --- | --- | --- |
| Original | 98.69 | 1.31 | 43.3 | 118 | 50462 | 2.9 |
| 8MPa | 99.35 | 1.31 | 42.9 | 124 | 48079 | 3.2 |
| 15MPa | 96.73 | 1.47 | 43.1 | 124 | 40799 | 3 |
| 30MPa | 95.03 | 4.84 | 44.7 | 288 | 11332 | 2.6 |
| 8MPall | 99.35 | 2.61 | 42.9 | 133 | 44618 | 3.2 |
| All | 98.69 | 2.09 | 43.1 | 184 | 32547 | 3.4 |
| Reference | 99.02 | 4.41 | 43.1 | 575 | 22139 | 3.6 |

^*^The original means the MAG information for the original sample. The incubation pressures were further set as 8, 15, and 30 MPa and then 8 MPa accordingly to mimic the cold seep environments at different water depths (1). All means the pool of original, 8MPa, 15MPa, 30MPa, 8MPall samples together, Reference means the genomes from the reference (2). The taxonomy of these ANME MAGs is d__Archaea; p__Halobacteriota; c__Methanosarcinia; o__Methanosarcinales; f__Methanocomedenaceae; g__Methanocomedens; s__Methanocomedens sp014237145 according to GTDB reference data version R207.

Table S2. The appearance of enzymes in five ANME MAGs

Table S3. List of the 77 cold seep metagenomic datasets to analyze the PCL and BCL encoding genes distribution in cold seeps.

| **JGI Sample Number** | **NCBI Acession Number** |
| --- | --- |
| 3300001685 | ERR514702 |
| 3300001749 | ERR514703 |
| 3300002180 | ERR514704 |
| 3300002481 | ERR514705 |
| 3300002532 | ERR514706 |
| 3300002966 | ERR514707 |
| 3300006972 | SRR1555744 |
| 3300007533 | SRR1555748 |
| 3300007999 | SRR1633224 |
| 3300008000 | SRR1633225 |
| 3300008019 | SRR1633226 |
| 3300009010 | SRR1971619 |
| 3300009039 | SRR1971620 |
| 3300009082 | SRR1971621 |
| 3300009085 | SRR1971622 |
| 3300009111 | SRR1971623 |
| 3300009149 | SRR1971624 |
| 3300009150 | SRR1971625 |
| 3300009499 | SRR1976948 |
| 3300009503 | SRR1977249 |
| 3300009529 | SRR1977296 |
| 3300009591 | SRR1977304 |
| 3300009713 | SRR1977357 |
| 3300009772 | SRR1977365 |
| 3300009775 | SRR1995427 |
| 3300009778 | SRR1995428 |
| 3300010264 | SRR1995429 |
| 3300010302 | SRR2132206 |
| 3300010317 | SRR2133563 |
| 3300010319 | SRR2133565 |
| 3300010324 | SRR2133566 |
| 3300010328 | SRR2133847 |
| 3300010330 | SRR2133850 |
| 3300010332 | SRR2133851 |
| 3300001854 | SRR2133852 |
| 3300002171 | SRR3715698 |
| 3300005860 | SRR3715733 |
| 3300006636 | SRR5396644 |
| 3300009590 |  |

Table S4. Bacteria strains and plasmids used in this study.

| **strains or plasmid** | **relevant characteristics** | **reference or source** |
| --- | --- | --- |
| **strains** |  |  |
| *E. coli* DH5α | *supE44 ΔlacU169* (*φ*80dlacZ *ΔM15*) *hsdR17 recA1 endA1 hsdR17 thi-1 gyrA96 relA1* | Novagen |
| *E. coli* BL21(DE3) | *F^-^ ompT hsdSB* (*Rb-mB-*) *gal* (*λc I 857 ind1 Sam7 nin5 lacUV5 T7gene1*) *dcm* (*DE3*) | Novagen |
| **plasmids** |  |  |
| pET-28a (+) | Kan^r^, overexpression vector | Novagen |
| pET-28a-*bcl*_ANME_ | BCL_ANME_ encoding gene cloned into pET-28a | this study |
| pET-29a-*pcl1*_ANME_ | PCL1_ANME_ encoding gene cloned into pET-28a | this study |
| pET-30a-*pcl2*_ANME_ | PCL2_ANME_ encoding gene cloned into pET-28a | this study |

The information of the Supplementary Tables:

Sheet1. The information of the 62 ANME MAGs.

Sheet2. List of the distributed PCL information in 77 cold seeps.

Sheet3. List of the distributed BCL information in 77 cold seeps.

References

1. Yang S, Lv Y, Liu X, Wang Y, Fan Q, Yang Z, Boon N, Wang F, Xiao X, Zhang Y. 2020. Genomic and enzymatic evidence of acetogenesis by anaerobic methanotrophic archaea. Nature communications 11:1-11.

2. Wang F-P, Zhang Y, Chen Y, He Y, Qi J, Hinrichs K-U, Zhang X-X, Xiao X, Boon N. 2013. Methanotrophic archaea possessing diverging methane-oxidizing and electron-transporting pathways. The Isme Journal 8:1069.
